# Supplementary material for: UFM: Unified feature matching pre-training with multi-modal image assistants
Source: PLoS One. 2025 Mar 31;20(3):e0319051. doi: 10.1371/journal.pone.0319051 (PMC11957561; doi:10.1371/journal.pone.0319051)
Supplement: Attachment — Submitted filename: Response to Editors and Reviewers Comments2024_12_20.docx [file pone.0319051.s001.docx]

**Response to Editors and Reviewers Comments**

Dear Editors and Reviewers,

Thank you so much for your comments about our manuscript entitled “UFM: Unified Feature Matching Pre-training with Multi-Modal Image Assistants” (PONE-D-24-47780). We carefully revised this manuscript according to these comments from the editors and reviewers to further improve this manuscript. All the comments are copied in blue color, and our responses are provided in black. In the latest revision of the paper, important changes are labeled by red font. We carefully revise the whole paper to improve the English expression of this paper. We do appreciate your serious consideration for our changes in this version.

Sincerely yours,

Yide Di

[ghostdyd@126.com](mailto:ghostdyd@126.com)

Yun Liao

LiaoYun@ynu.edu.cn

**Reviewer 1:** 
This paper introduces UFM (Unified Feature Matching), a pre-trained model designed to handle feature matching across multiple image modalities. The key innovation is the Multi-Modal Image Assistant (MIA) Transformer architecture that enables both same-modality and cross-modality feature matching through a shared parameter space. The authors propose a staged pre-training strategy and data augmentation techniques to address challenges with imbalanced modal datasets. The work is evaluated across various datasets including optical, SAR, NIR, SWIR, and medical images, demonstrating competitive performance against specialized methods.

Strengths

-Novel MIA Transformer architecture that effectively handles both same-modality and cross-modality matching

-Good solution to the modal imbalance problem through staged pre-training

Comprehensive Evaluation

-Addresses a real-world need for unified feature matching across modalities

Major issues:

(1) Data augmentation strategy needs more detailed explanation.

**Response 1):** Thank you so much for your comments. We have carefully modified the schema of the data enhancement strategy and added the explanation. The specific modifications are as follows:

As shown in Fig. 3, given an image pair $\mathrm{Image}_{a}$ and $\mathrm{Image}_{b}$, a sequence of data augmentation procedures is initially applied. Subsequently, these enriched image pairs are utilized to produce a comprehensive pixel matching label GT matrix. The augmentation process includes mirroring, flipping, and rotating the input images, significantly enhancing the diversity within the dataset. In addition, the processed image is randomly cropped. Finally, random noise is added to the cropped block map, and some pixels are randomly masked.

2) Transition between the pre-training stages is not clearly described.

**Response 2):** Thank you so much for your suggestion. We have revised the diagram of the stage transformation of pre-training and added more explanations. The specific modifications are as follows:

Presented in Fig. 4, our proposed staged pre-training strategy aims to enhance the image matching model’s performance by leveraging a large-scale image dataset from the same modality. The pre-training is divided into 3 stages: (1) pre-train the general FFN, (2) pre-train all X-X assistants, and (3) pre-train all X-Y assistants. In stage 1, given that optical images provide a rich source of feature matching data, we initially conduct pre-training on multi-head attention and the generic feedforward network (FFN) using a substantial collection of pure optical images. In stage 2, We pre-train feature matching for all of the same modal images (X-X). The pre-training of all modal images (X-X, Y-Y, Z-Z···) in the stage 2 can be performed in parallel. We freeze multi-head self+cross attention at this stage, which greatly improves the efficiency of training. In stage 3, we further pre-train the feature matching of all cross-modal images (X-Y) on the basis of stage 2. At this stage, we adjusted all the attention and corresponding FFNS to maximize cross-modal matching. The three FFNS (X-X, Y-Y, X-Y) corresponding to the two modal images are pre-trained simultaneously.

3) Need for more extensive failure cases and analysis to help readers know the limitation.

**Response 3):** Thanks for your suggestion, we have added a section dedicated to the limitations of UFM, as follows:

## 4.8 limitation

Although UFM has been shown to deliver good matching results in most cases, it also has some limitations. As illustrated in Fig. 13, UFM struggles to achieve accurate matching when dealing with multi-modal images that have very few textures. This is a common challenge for feature matching methods, and other approaches also face difficulties in such scenarios. While UFM has proven to be highly generalizable, its pre-training becomes less effective when confronted with unseen modal images. In such cases, as with other methods, it is necessary to train the model on the entire dataset to achieve accurate matching results.

4) Figures captions are mostly lack sufficient details and explanations.

Thank you very much for your advice. We have modified all the picture captions according to your suggestions, and the revised picture captions are as follows (Figures 3 and 4 have been shown above):

5) How to address with or make sure the training stability across different modalities?

Image feature matching typically involves matching a pair of images. However, when there are multiple modalities of an image representing the same scene—such as three modalities: X, Y, and Z. we need to match images from any two of these modalities. Specifically, this means matching X-Y, X-Z, and Y-Z pairs. These three cases correspond to different modal matching, as shown in the third stage of Figure 4.

6) Few past works in feature matching are missing in literature review, including [Maskflownet: Asymmetric feature matching with learnable occlusion mask][Superthermal: Matching thermal as visible through thermal feature exploration][CorMatcher: A corners-guided graph neural network for local feature matching], etc

**Response 6):** Thank you so much for your suggestion and it is very helpful. We read the papers you suggested carefully and found them very helpful. We cited all the papers you suggested and went on to cite some other valuable papers.

Thank you again for your comments. And we sincerely hope our modifications will make you be satisfied in this version.

**Reviewer 2:** 
1) Page 3, contribution 3: Please revise the sentence "Remarkably, it has exhibited remarkable performance."

**Response 1):** Thank you so much for your suggestion. We have revised the contribution on page 3. The revised content is as follows:

3. Our proposed method demonstrates the ability to handle feature matching tasks for images of the same modal with significant disparities in sensor poses, as well as for feature matching tasks between images of different modals.

2) Method description could benefit from more clarity in the architecture overview. At times, it is not clear to me which components relate to the UFM and which to the MIA. This could be reinforced with small augmentations to Fig. 2.

**Response 2):** Thank you so much for your suggestion and it is very helpful. We modified Figure 2 according to your request. The revised figure 2 is as follows:

3) Method could also use clarity on the nature of the model's outputs. There's a per-element mapping matrix (GT_matrix) which is more or less described, but there are also references to a "heatmap" which first appears on page 8 in discussion of the loss function.

**Response 3):** Thank you so much for your suggestion and it is very helpful. We have modified this part in detail according to your request. The contents of the modification are as follows:

Following [48], we used the epipolar loss. The epipolar constraint states that $j^{T}F_{i}=0$ holds if $i$ and $j$ are truly matched, where $F_{i}$ can be interpreted as the epipolar line corresponding to $i$ in $I_{b}$. The epipolar loss is defined as the distance between the predicted corresponding position and the ground-truth epipolar line:

$L_{ep}\left( i \right)=dist(h_{1\to2}\left( i \right),F_{i})$,

where $h_{1\to2}\left( i \right)$ is the predicted correspondence in $I_{b}$ for the point $i$ in $I_{b}$, and $dist(\cdot,\cdot)$ is the distance between a point and a line.

The epipolar loss itself only encourages the predicted match to lie on the epipolar line rather than close to the ground truth correspondence. We also need to introduce a cycle consistency loss to encourage the forward and backward mapping of a point to be spatially close to itself:

$L_{cy}\left( i \right)={|\left| h_{2\to1}\left( h_{1\to2}\left( i \right) \right)-i \right||}_{2}$.

For a pair of enhanced images $I_{a}$ and $I_{b}$, the dense feature descriptors extracted by MIA transformer are defined as $M_{1}$ and $M_{2}$. To compute the correspondence for a query point $i$ in $I_{a}$, we correlate the feature descriptor at $i$, denoted by $M_{1}$($i$), with all of $M_{2}$. A 2D pixel location distribution of $I_{b}$ is obtained, indicating the probability corresponding to each location and $i$ in $I_{a}$. The probability distribution can be expressed as $p\left( x | i,M_{1},M_{2} \right)$. A single 2D match can then be computed as the expectation of this distribution:

$$\hat{j}=h_{1\to2}\left( i \right)=\sum_{x\in I_{b}} x\cdot\frac{exp(M_{1}\left( i \right)^{T}M_{2}(x))}{\sum_{y\in I_{b}} exp(M_{1}\left( i \right)^{T}M_{2}(y))}$$

where y varies over the pixel grid of $I_{b}$.

The loss at each point is re-weighted using the total variance $\sigma^{2}(i)$ as an uncertainty measure. The loss function for fine matching is the weighted sum of the epipolar and cycle consistency loss of the n sampled query points:

${Loss}_{f}=\sum_{m=1}^{n} \frac{1}{\sigma^{2}(i^{m})}[L_{ep}\left( i^{m} \right)+\lambda L_{cy}\left( i^{m} \right)]$.

4) Given the non-linear intensity relationships across imaging modalities, I find it curious that all data augmentations are purely geometric. Were any intensity augmentations considered?

**Response 4):** Thank you so much for your suggestion and it is very helpful. We have modified the data augmentation module according to your suggestion, and the modified content is as follows:

As shown in Fig. 3, given an image pair $\mathrm{Image}_{a}$ and $\mathrm{Image}_{b}$, a sequence of data augmentation procedures is initially applied. Subsequently, these enriched image pairs are utilized to produce a comprehensive pixel matching label GT matrix. The augmentation process includes mirroring, flipping, and rotating the input images, significantly enhancing the diversity within the dataset. In addition, the processed image is randomly cropped. Finally, random noise is added to the cropped block map, and some pixels are randomly masked.

5) I am slightly unclear about the training sequence for the feature assistants. The presented training steps seem to be three stages of pretraining: 1) Generic FFN Pre-training, 2) train all X-X assistants, 3) train all X-Y assistants. If this is correct, then I think the text and Fig. 4 could clarify that these are three stages of pretraining. Since the example only considers two modalities (Opt, SAR), how does this method change for greater numbers of modalities? It seems like all X-X assistants can be trained in parallel, but that all X-Y assistants might need to be trained simultaneously.

**Response 5):** Thank you very much for your advice, your understanding of the pre-training phase is correct. We modified the figure of the pre-trained as you suggested and added some explanations. The details are as follows:

Presented in Fig. 4, our proposed staged pre-training strategy aims to enhance the image matching model’s performance by leveraging a large-scale image dataset from the same modality. The pre-training is divided into 3 stages: (1) pre-train the general FFN, (2) pre-train all X-X assistants, and (3) pre-train all X-Y assistants. In stage 1, given that optical images provide a rich source of feature matching data, we initially conduct pre-training on multi-head attention and the generic feedforward network (FFN) using a substantial collection of pure optical images. In stage 2, We pre-train feature matching for all of the same modal images (X-X). The pre-training of all modal images (X-X, Y-Y, Z-Z···) in the stage 2 can be performed in parallel. We freeze multi-head self+cross attention at this stage, which greatly improves the efficiency of training. In stage 3, we further pre-train the feature matching of all cross-modal images (X-Y) on the basis of stage 2. At this stage, we adjusted all the attention and corresponding FFNS to maximize cross-modal matching. The three FFNS (X-X, Y-Y, X-Y) corresponding to the two modal images are pre-trained simultaneously.

Since the example only considers two modalities (Opt, SAR), how does this method change for greater numbers of modalities?

Image feature matching typically involves matching a pair of images. However, when there are multiple modalities of an image representing the same scene—such as three modalities: X, Y, and Z. we need to match images from any two of these modalities. Specifically, this means matching X-Y, X-Z, and Y-Z pairs. These three cases correspond to different modal matching, as shown in the third stage of Figure 4.

6) The experiments do not describe when fine-tuning was deployed and what data was used for model fine-tuning.

**Response 6):** Thank you so much for your suggestion. We have made the modification according to your suggestion, and the modified content is as follows:

## 3.4 Fine-Tuning on Different Feature Matching Tasks

After comprehensive pre-training, UFM only requires fine-tuning on the corresponding dataset to achieve excellent matching results. For instance, when dealing with a brand-new multimodal dataset, other methods typically need to be pre-trained on the entire training set. In contrast, UFM usually requires only about 1/10 of the training data for fine-tuning to deliver superior matching performance. This fine-tuning process includes both within-modal feature matching and cross-modal image feature matching. This critical stage ensures that the model effectively adapts to the unique characteristics and complexities of the matching task.

## 4.1 Pre-Training Setup

Our pre-training data mainly consists of these datasets: MegaDepth [44], ScanNet [45], YFCC100M [46], OSCD [47], MRSI [48], MRSIs [33], DIRSIG [49], MS-SAR LCZ [50], Retina [51], BrainWeb [52], VIS–NIR [53], WxBS [54] and image–paint [55]. There are about 2 million image pairs in the pre-training data. After pre-training, we only need to use about 1/10 of the training set of the corresponding dataset for fine-tuning.

## 4.2 Estimation on Same-Modal Images Feature Matching

In the feature matching evaluation of images from the same modality, we mainly verify the feature matching between images with large camera pose differences. As shown in Fig. 7, We compare the feature matching of different methods on Hpatches [56], InLoc [57] and Aachen Day-Night v1.1 [58] datasets. The InLoc and Aachen Day-Night v1.1 datasets are just the datasets for testing and do not have any training data. We fine-tuned on the Hpatches dataset using 1/10 of the training data.

## 4.3 Estimation on Different-Modals Images Feature Matching

In the evaluation of feature matching between images of different modals, seven datasets were used for testing. As shown in Fig. 8, the data sets used for the test are: SEN 12 MS dataset [72], RGB-NIR Scene dataset [53], WHU-OPT-SAR dataset [73], Optical-SAR dataset [74], BrainWeb dataset [52], NYU-Depth V2 dataset [75] and UV-Green dataset [76]. We fine-tuned on these datasets using 1/10 of the training data. UFM can handle most multi-modal feature matching problems. This test experiment mainly covers optical images, SAR images, NIR images, SWIR images, depth images, UV images, green images and medical images.

7) It's not clear to me how Homography estimation is a valuable tasks for the different-modals same-scene feature matching. My understanding was that these image pairs are already close to alignment, as shown in Fig. 8. Is some augmentation imposed?

9) Fig. 11 and Fig. 12 - Please add more description to the caption, specifically mentioning that only matches with <1px error are plotted.

10) For Fig 12, it actually isn't visually clear that your method is superior. Perhaps consider a plot of the number of matches with <1px error across methods?

**Response 7&9&10):** Thank you so much for your suggestion. Thank you very much for your advice. We did augment the images, but forgot to show the augmented images. We modified Figures 11 and 12 as you suggested. Here's the change:

8) In the beginning of sec 4.4, you simply state that "the correct matches are represented by lines," but do not define a "correct match." What is the error margin for a "correct match"? Later, you state that it's <1px error, but not here.

**Response 8):** Thank you so much for your suggestion. We have made the modification according to your suggestion, and the modified content is as follows:

**The images of the same modal.** As illustrated in Fig. 11, in order to observe the matching effect of different methods more intuitively, the matches with less than 1 pixel error are represented by lines. To save space, we only show the matching results of the four methods with the best results. Compared with other methods, UFM has more correct matching lines, which qualitatively proves that UFM algorithm has a good effect on image feature matching of the same modal.

**The images of different modals.** The results of multi-modal image feature matching are generated, and the results of the four methods with the best results are presented in Fig.12. The datasets from top to bottom are: SEN 12 MS dataset, RGB-NIR Scene dataset, WHU-OPT-SAR dataset, Optical-SAR dataset, BrainWeb dataset, NYU-Depth V2 dataset and UV-Green dataset. In order to clearly compare the matching effect of different methods, we only connected the matching points with pixel error less than 1 pixel. Compared with other methods, the UFM algorithm obtains more correct matching connections, which qualitatively proves the competitiveness of the UFM algorithm in dealing with multi-modal image feature matching.

Thank you again for your valuable suggestions. Moreover, we do appreciate your serious consideration for our corrections in this version.

**Reviewer 3:**

 The authors propose a UFM model, leveraging MIA Transformers, a novel approach to unified feature matching across diverse modalities.

Strengths: The experiments are very extensive by including a set of datasets and comparisons with state-of-the-art methods. The methodology is detailed in explaining the architecture, pre-training strategies, and data augmentation. Finally, the results, especially in comparison with existing methods, substantiate the model's effectiveness and generalizability.

Weaknesses:
1) fig 5 and fig 6 could be improved with correcting the subscripts and defining what '...' means or in what area is including the '...'

**Response 1):** Thank you so much for your suggestion. We have made the modification according to your suggestion, and the modified content is as follows:

2) while the performance gains are evident, I would like to see a statistical analysis to confirm the significance observed differences, such as p-values or confidence intervals.
3) the absence of variability measures makes it difficult to assess the consistency and robustness of results. Such as, standard deviations or error bars.

**Response 2&3):** Thank you very much for your advice. Due to the particularity of the dense matching problem, some traditional evaluation methods are not very applicable. We found a suitable evaluation method by reviewing a large number of papers: evaluating the distance error of matching points for different methods. Here's the relevant content we added:

4.5 Estimation on the distance error test of feature matching

Although the lines of matched points with pixel error less than 1 are shown in Section 4.4, it is not possible to show the specific error value for each pixel. To further objectively evaluate the matching accuracy of different methods, we calculated the average distance error of matching points with horizontal and vertical pixel errors less than 1. The total error is computed based on both the horizontal and vertical pixel errors, and its value may exceed 1. In this experiment the horizontal distance error is defined as $H_{rmse}$, the vertical distance error is defined as $V_{rmse}$and the total distance error on the image is defined as ${HV}_{rmse}$. All errors are measured in pixels. The functions $H_{rmse}$, $V_{rmse}$, and ${HV}_{rmse}$ are computed as:

$$H_{rmse}=\sqrt{\frac{1}{N}\sum_{i} {(a_{i}^{x}-a_{i}^{y})}^{2}}$$

$$V_{rmse}=\sqrt{\frac{1}{N}\sum_{i} {(b_{i}^{x}-b_{i}^{y})}^{2}}$$

$${HV}_{rmse}=\sqrt{\frac{1}{N}\sum_{i} (\left( a_{i}^{x}-a_{i}^{y} \right)^{2}+\left( b_{i}^{x}-b_{i}^{y} \right)^{2})}$$

where $(a_{i}^{x},b_{i}^{x})$ denotes the coordinates of the matching points in the X-modal image, $(a_{i}^{y},b_{i}^{y})$ indicates the coordinates of the matching point in the Y-modal image. N denotes the total number of matched points.

## As shown in Table 6, UFM consistently achieves the smallest distance error in most cases, outperforming other methods. The experiments further demonstrate that UFM not only identifies the maximum number of matching points with an error of less than 1 pixel, but also ensures that the error of the obtained matching points remains very small.

4) There is little discussion on the computational cost of the UFM model compared to competing methods. Including this would give a more complete picture of its practical applicability.

**Response 4):** Thank you so much for your suggestion. According to your suggestion, we have added the experiment content of the computational cost, which is as follows:

## 4.6 Evaluation of computational cost

To objectively evaluate the computational cost of the UFM algorithm, we compare the matching speed and resource requirements across different methods. In this experiment, the matching methods are tested on the RGB-NIR Scene dataset using an RTX 3090 GPU and an Intel i7-11700 processor. Matching speed is measured in frames per second (FPS), and resource usage is assessed in terms of storage requirements (MB). A higher FPS indicates faster inference, while lower storage values reflect reduced resource usage. As shown in Table 7, UFM achieves the fastest matching speed. Among the methods compared, UFM, LoFTR, and FeMIP are semi-dense matching techniques, while the remaining methods are sparse matching approaches. Generally, semi-dense methods offer higher matching accuracy but tend to demand more resources. Notably, UFM requires the least resources among the semi-dense methods.

5) Quantitative are strong, but there is a lack of qualitative evaluation of feature matching performances. More visual examples would help.

**Response 5):** Thank you so much for your suggestion. According to your suggestion, we have added the experiment content of the qualitative evaluation, which is as follows:

6) While UFM is presented as highly generalizable, more empirical evidence or theoretical discussion on its adaptability to less common modalities would strengthen this claim.

**Response 6):** Thank you so much for your suggestion. we have added a section dedicated to the limitations of UFM, as follows:

## 4.8 limitation

Although UFM has been shown to deliver good matching results in most cases, it also has some limitations. As illustrated in Fig. 13, UFM struggles to achieve accurate matching when dealing with multi-modal images that have very few textures. This is a common challenge for feature matching methods, and other approaches also face difficulties in such scenarios. While UFM has proven to be highly generalizable, its pre-training becomes less effective when confronted with unseen modal images. In such cases, as with other methods, it is necessary to train the model on the entire dataset to achieve accurate matching results.

7) while the ablation studies are valuable, deeper interpretation of the interactions between the model components (data augmentation and MIA transformers interaction) to determine the performance boost.

**Response 7):** Thank you so much for your suggestion. According to your suggestion, we have modified the ablation experiment. The revised content is as follows:

4.7 Ablation Study

To fully assess the role of different modules in UFM, seven variants were designed. In the 7th variant, pre-training is omitted, and the model is trained using the entire training dataset. As shown in Tables 8 and 9, we conduct ablation experiments on feature matching using images from the same modal and from different modals, respectively. The results of these experiments demonstrate that UFM outperforms all variants in feature matching. The combination of data augmentation and the MIA transformer is particularly effective, with the Assistant FFN in the MIA transformer having the most significant impact. The Generic FFN has a lesser effect on the matching performance. Data augmentation, Assistant FFN, and fine-tuning have a substantial influence on feature matching across different modals, while their impact on same-modal feature matching is relatively smaller. Although the 7th variant is trained on the full dataset, it still underperforms compared to the complete UFM, highlighting the necessity of the pre-training and fine-tuning mechanisms we have designed.

Thank you again for your comments. And we sincerely hope our modifications will make you be satisfied in this version.
